# A review on the controlled preparation, structure–property relationship and application progress of multifunctional metal nanoparticle-based polymer nanocomposites

**DOI:** 10.1039/d6ra02629j

**Published:** 2026-07-02

**Authors:** Zhen Gou

**Affiliations:** a College of Material Science and Engineering, Northeast Forestry University Harbin 150040 China GOUZ123@nefu.edu.cn

## Abstract

Metal nanoparticle-based polymer nanocomposites represent a versatile materials platform for integrating electrical, optical, thermal, catalytic, antimicrobial, and mechanical functionalities into lightweight, processable polymer matrices. However, their performance cannot be rationalized by metal identity or filler loading alone. Instead, it is governed by coupled structural descriptors, including nanoparticle size and morphology, dispersion state, interparticle spacing, interphase chemistry, network topology, and structural evolution under service conditions. This review summarizes recent advances in the controlled preparation, descriptor–property relationships, and application development of multifunctional metal nanoparticle–polymer nanocomposites from a descriptor-driven perspective. First, representative metal nanoparticles and polymer matrices are introduced and key concepts such as interphase effects, percolation behavior, and quantitative metrics for multifunctionality are outlined to establish a unified framework. Second, fabrication strategies, including *in situ* formation, *ex situ* incorporation, interfacial assembly, advanced patterning, green processing, and scalable manufacturing, are critically compared in terms of their capability to regulate key structural features. Third, the relationships among morphology, interphase-mediated transport, multifunctional coupling, predictive modeling, and long-term reliability are discussed to elucidate performance-limiting factors and design rules. Finally, applications in flexible and printed electronics, wearable sensors, energy conversion and storage, electromagnetic interference shielding, and catalytic systems are surveyed. In contrast to reviews organized by specific metals or individual applications, this review emphasizes transferable structural descriptors and cross-system performance trade-offs. Future progress will require standardized reporting, data-enabled structure–property mapping, scalable processing, and reliability-oriented design under realistic operating conditions.

## Abstract 2

This review presents a descriptor-driven framework linking morphology, dispersion, interphase, and topology to multifunctionality, trade-offs, design rules, scalability, reporting, and reliability.

## Graphical abstract

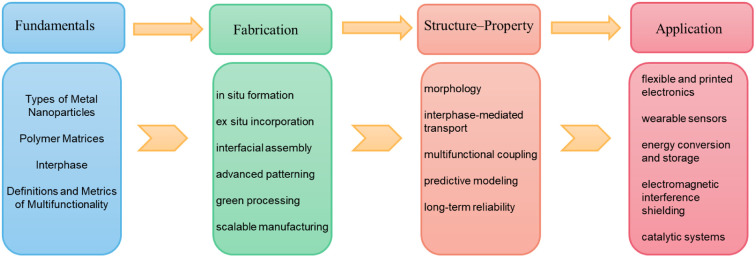

## 1 Introduction

Polymer nanocomposites have attracted sustained interest because nanoscale fillers can markedly alter the mechanical, electrical, thermal, barrier, and other functional properties of polymer matrices at relatively low loadings. Conventional nanofillers including nanoclay, carbon nanotubes, graphene, and related carbon-based materials have established key design principles in the field. Nanoclays, particularly layered silicates like montmorillonite are widely utilized for their ability to enhance stiffness, flame retardancy, and barrier properties through intercalation and exfoliation mechanisms.^1–4^ In contrast, carbon nanotubes and graphene possess exceptional intrinsic electrical and thermal conductivities, which have facilitated their extensive integration into polymer matrices to engineer multifunctional composites with enhanced mechanical strength, self-healing capabilities, and advanced sensing functionalities. Studies have shown that the self-healing microcapsules encapsulating carbon nanotubes (DCPD-CNT-UF) possess better thermal stability, higher electrical conductivity, and superior tensile strength, while maintaining the self-healing efficiency.^5–9^ Nonetheless, persistent limitations remain. Nanoclay is generally ineffective when strong electrical or thermal functionality is required. Carbon nanotubes and graphene often face high cost, strong agglomeration, limited compatibility with many polymer matrices, and narrow processing windows, particularly at high loadings. Even with surface functionalization, agglomeration and viscosity increases can constrain scalable processing and compromise reproducibility. These challenges have driven growing interest in metal nanoparticle-based polymer nanocomposites.

Compared to conventional reinforcing fillers, the diversity of metal nanoparticles offers a broader functional design space for polymer composites. Depending on their type, size, morphology, and surface state, metal nanoparticles can endow polymer matrices with properties such as electrical conductivity, photothermal conversion, catalytic activity, and antibacterial effects. Silver nanostructures are commonly used to construct conductive networks and antibacterial interfaces. Gold nanoparticles are suitable for plasmonic sensing and photothermal conversion. Copper-based nanoparticles, due to their lower cost, hold potential for forming conductive pathways. Platinum and palladium nanoparticles offer excellent catalytic and electrocatalytic activities. However, metal nanoparticles cannot simply be regarded as superior fillers. Although copper nanoparticles are low-cost and highly conductive, they are prone to oxidation, leading to conductivity degradation and interface instability. Silver nanoparticles combine high conductivity with strong antibacterial activity but also present issues such as ion migration, cytotoxicity, and environmentally induced performance drift. Gold nanoparticles exhibit outstanding chemical stability and optical activity, yet their high cost limits large-scale applications. Platinum and palladium nanoparticles demonstrate superior catalytic performance but face constraints from high cost, surface poisoning, and long-term deactivation. Thus, the practical value of metal nanoparticles does not lie solely in their intrinsic properties, but rather in whether these properties can be translated into stable, accessible, reproducible, and processable composite structures.

The ultimate performance of these nanocomposites depends not only on the metallic phase but also on the polymer matrix, the interface between the two phases, and the microstructure formed during processing. The polymer matrix is not merely an inert carrier. It plays an active role in structural formation and functional modulation by influencing nanoparticle localization, confinement, migration, and long-term stability through its chain dynamics, phase behavior, free volume distribution, and network architecture. Kumar and Krishnamoorthy^10^ emphasized that the properties of nanocomposites should be understood within a coupled framework involving structure, phase behavior, and interfacial interactions, rather than solely based on composition. Consequently, even identical metal nanoparticles may exhibit different responses when embedded in different polymer matrices. Although the concept of active control *via* interfaces and matrices is gaining increasing attention, a significant portion of research still focuses primarily on the synthesis, characterization, and intrinsic properties of metal nanoparticles themselves, simplifying the role of polymers to that of a mechanical support medium, without deeply exploring how the molecular structure and segmental dynamics of the matrix actively shape nanoparticle evolution and final functional outcomes. For instance, in their study on cobalt nanoparticles synthesized in a polystyrene matrix, Nasralla *et al.*^11^ began with the magnetic and optical responses of the cobalt nanoparticles themselves, while regarding styrene mainly as a stable chemical environment.

Despite such remarkable progress, there are still some significant limitations in the current literature. Firstly, many existing studies and reviews are still organized around a single material category, a single filler type, or a single target application, which makes it difficult to compare how different preparation routes adjust common structural descriptors in different systems.^10,12–17^ Aliyeva *et al.*^18^ focused on metal–polyolefin nanocomposites, Wawrzyńczak *et al.*^19^ limited their review to biodegradable polymer matrices, and Jamwal *et al.*^20^ specifically addressed HDPE composites reinforced with a single conductive filler. Secondly, although numerous reports have demonstrated remarkable electrical, optical, photothermal, antibacterial, catalytic or shielding properties, the structural–performance relationships behind them are often discussed in fragmented ways, with insufficient attention paid to interface chemistry, dispersion state, network topological structure, and service-induced structural evolution.^10,15–17,21–24^ Recently, Sharma *et al.*^25^ in their review on polymer-based nanocomposites pointed out that the progress in this field is still fragmented in filler-specific research and often lacks a unified mechanistic explanation. Thirdly, the actual multifunctionality is not merely the coexistence of several high values. Instead, it involves the trade-offs between conductivity, flexibility, transparency, heat conversion, biocompatibility, environmental stability, and scalability, but these trade-offs have not been fully integrated into the current review framework.^26–31^ Abdulraheem *et al.*^32^ discussed the multifunctionality of metal nanoparticle polymer composites, but tended to adopt an additive narrative logic and rarely integrated the coupling relationships among these properties, the trade-offs of this and that, into a unified analytical framework. Therefore, this field still lacks a transferable design logic that can systematically link metal nanoparticle types, polymer matrix selection, controllable manufacturing, structural organization, multifunctional coupling, and device-level reliability.

Based on this, this paper regards metal nanoparticle–polymer nanocomposites as a mixed system governed by structural descriptors, rather than a simple combination of metal fillers and polymer matrix. The goal of this paper is to clarify how controllable preparation, structural organization, interface regulation, and multifunctional coupling jointly determine the application-related properties. To this end, representative metal nanoparticles and polymer matrices are introduced, and the influence of interfaces, dispersion states, and percolation behaviors on functional expression is discussed. Second, starting from the regulatory ability of structural descriptors, the main strategies such as *in situ* formation, exogenous incorporation, interface assembly, patterning processing, green preparation, and large-scale manufacturing are compared. Subsequently, the structure–performance relationship around morphology regulation, interface-mediated transport, multifunctional trade-off, multi-scale characterization, theoretical modeling, and long-term stability analysis is discussed. Finally, from the structural requirements rather than isolated performance indicators, the development of application fields such as flexible electronics, wearable sensing, antibacterial and biomedical interfaces, energy-related systems, electromagnetic shielding, and catalysis is summarized. By organizing existing research with transferable structural descriptors and key performance trade-offs as the main lines, this paper aims to provide a more systematic reference for the rational design, performance optimization, and practical transformation of multifunctional metal nanoparticle–polymer nanocomposites.

## 2 Fundamentals and conceptual framework

**Fig. 1:**
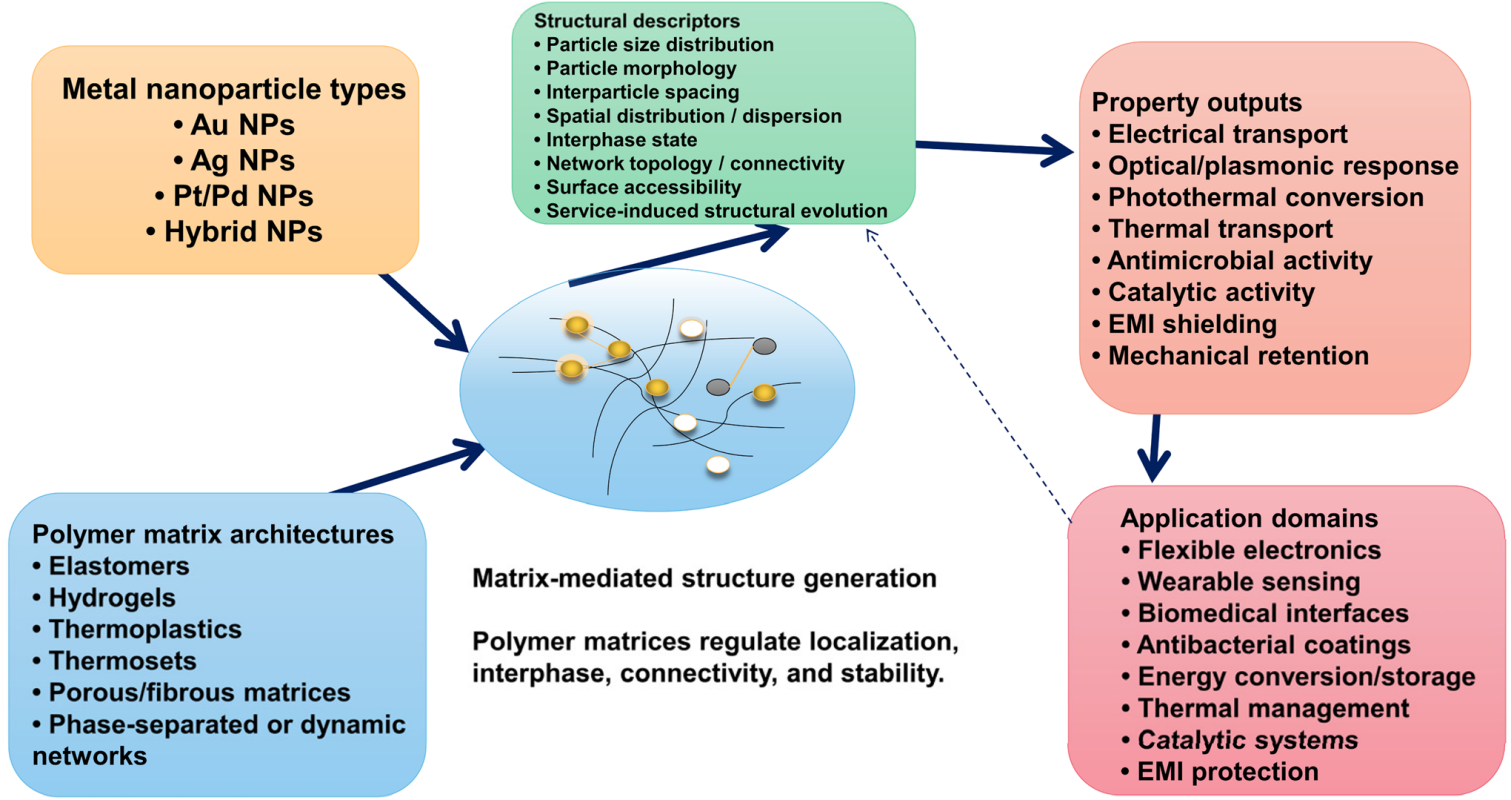
Descriptor-based framework of metal nanoparticle–polymer nanocomposites.

Multifunctional metal nanoparticle based polymer nanocomposites constitute a class of hybrid materials in which the electronically, optically, catalytically, magnetically, and biologically active characteristics of metallic nanostructures are integrated with the low density, processability, mechanical compliance, and structural tunability of polymer matrices. Their macroscopic properties do not arise from a simple superposition of the intrinsic attributes of metals and polymers. Rather, they are determined by the manner in which metal nanoparticles are spatially organized within the polymer phase, by the physicochemical character of the interfacial region, and by the evolution of such structures under service conditions. In conductive and electromagnetic systems, the dominant variables include the filler volume fraction *ϕ*, the percolation threshold *ϕ*_c_, the effective interparticle gap *g*, and the barrier characteristics that govern charge transport across interphases. In plasmonic and photothermal systems, the decisive factors are resonance behavior, local electromagnetic field confinement, and coupling between metallic nanostructures and the dielectric environment of the surrounding polymer. In antimicrobial and biointerfacing systems, functional response is further shaped by oxidation accessibility, ion release kinetics, diffusion resistance, surface exposure, and biosafety constraints. A coherent theoretical framework for this field must therefore be established from four interdependent dimensions, namely metal nanoparticle type, polymer matrix architecture, interphase governed dispersion and percolation behavior, and operational definitions together with evaluation metrics of multifunctionality (Fig. 1).

### 2.1 Types of metal nanoparticles for multifunctional nanocomposites

In polymer nanocomposites designed for multifunctional performance, metal nanoparticles should be understood as active structural nodes rather than inert inclusions. The metal species defines the accessible functional channel, whereas the eventual response is governed by how that channel is expressed within a polymer mediated microstructure. Parameters such as particle diameter *d*, morphology, exposed surface state, aggregation state, and interparticle spacing collectively determine whether the intrinsic electronic or chemical characteristics of the metallic phase can be translated into measurable and stable performance. Azharuddin *et al.*^33^ systematically reviewed the biomedical application landscape of noble metal nanoparticles and showed that even identical metallic compositions may trigger markedly different physicochemical or biological responses when structural state and interfacial environment are altered. That conclusion is directly relevant to polymer nanocomposites. In these systems, the polymer host not only accommodates nanoparticles, but also modulates local dielectric properties, transport pathways, confinement effects, and interfacial accessibility. Metal type thus specifies the upper bound of functionality, whereas composite structure determines whether that functionality remains accessible, reproducible, and application relevant.

#### 2.1.1 Plasmonic optics and energy conversion of noble-metal nanoparticles

The multifunctionality of noble metal nanoparticles is closely associated with collective oscillations of conduction electrons. Jain *et al.*^34^ showed that noble metal nanoparticles display pronounced optical absorption and photothermal responses at the nanoscale, with localized surface plasmon resonance providing the fundamental mechanism. The resonance position and spectral line shape are strongly dependent on particle size, morphology, and the dielectric properties of the surrounding medium. Once interparticle gaps become sufficiently small, plasmonic coupling emerges and gives rise to localized electromagnetic hot spots, which markedly increase field intensity and local energy deposition. Jain *et al.*^35^ further reviewed plasmon enhanced effects in biosystems and demonstrated that resonance amplification is not a fixed material property, but a structurally contingent response governed by the degree of field localization and by local refractive index conditions. These observations acquire additional significance in polymer nanocomposites, because polymer matrices modify the dielectric environment, constrain nanoparticle spacing, and influence the routes through which absorbed energy is dissipated. Noble metal nanoparticles with similar compositions may therefore exhibit substantially different spectral responses and photothermal efficiencies when incorporated into different polymer hosts. These basic concepts are equally applicable to photoelectric conversion. In polymer solar cells, various plasmonic nanoparticles with different shapes and sizes have been integrated at different positions within OSC layers to enhance light absorption, promote the generation of electron–hole pairs, and enable the transport of free charge carriers, ultimately increasing the photocurrent in OSCs. Waketola *et al.*^36^ investigated how the shape and size of Au and Ag NPs affect the power conversion efficiency of OSCs when they are integrated into different device layers, and pointed out that smaller NPs enhance light absorption in the absorber layer due to local field enhancement, whereas larger NPs scatter light owing to their larger scattering cross sections. Tzounis *et al.*^37^ incorporated gold–silver core–shell nanoparticles into the active layer of P3HT:PCBM devices, using plasmonic nanoparticles to improve the power conversion efficiency of air-processed OPVs by 20.1%.

In energy-storage systems, particularly when used as supercapacitors, the introduction of metal nanoparticles can improve electronic conductivity, increase the electrochemically active interfacial area, and shorten ion and electron transport pathways, thereby ultimately enhancing device performance. Kyomuhimbo and Feleni^38^ summarized the applications of metal/polyaniline nanocomposites in supercapacitors and bistable memory devices. Çıplak and Yıldız^39^ prepared a gold nanoparticle-polyaniline binary nanocomposite, which exhibited high electrochemical performance with a specific capacitance of 292.2 F g^−1^ at a current density of 0.5 A g^−1^.

The sensing behavior of noble metal nanoparticles is equally dependent on structural context. Doria *et al.*^40^ summarized the principal signal transduction mechanisms in biosensing, including resonance shifts induced by refractive index perturbation, enhancement of scattering and Raman signals, and selective recognition enabled by surface functionalization. In polymer nanocomposites, however, these mechanisms require sustained surface accessibility and effective suppression of uncontrolled aggregation. If nanoparticle clustering proceeds without control, field enhancement is accompanied by spectral broadening, elevated background interference, and loss of selectivity. The optical functionality of noble metal nanoparticles in polymer media should therefore be interpreted not as an intrinsic consequence of composition alone, but as the outcome of structure dependent regulation of local field distribution and energy dissipation.

#### 2.1.2 Antimicrobial mechanisms and structural dependence of silver nanoparticles

Silver nanoparticles represent one of the most widely used antimicrobial nodes in polymer nanocomposites, yet their biological activity cannot be reduced to a single release-based mechanism. Bruna *et al.*^41^ summarized that the antimicrobial effect of silver nanoparticles is associated with Ag^+^ release, membrane damage, and oxidative stress, while smaller particle dimensions and higher surface activity may enhance efficacy but also increase reactivity and toxicological concern. Meher *et al.*^42^ further emphasized that biomedical suitability depends not only on composition, but also on surface chemistry, colloidal stability, and the regulatory role of the surrounding polymer with respect to release behavior. In polymer nanocomposites, the matrix governs water uptake, diffusion resistance, adsorption and desorption equilibria, and the accessibility of the nanoparticle surface. The polymer phase therefore controls the local concentration field of Ag^+^, the duration of interfacial exposure, and the extent to which oxidative reactions can proceed under physiological or microbially relevant conditions. Rodrigues *et al.*,^43^ drawing on proteomic evidence, highlighted the complexity of bacterial responses and made clear that minor variations in exposure pathway, release rate, and interfacial concentration may lead to substantial differences in antibacterial outcome. Antimicrobial performance in silver nanoparticle polymer nanocomposites must therefore be discussed through the coupled variables of oxidation accessibility, ion release kinetics, aggregation evolution, and diffusion limited transport, rather than through filler loading or particle size alone.

The principal redox pathway may be represented schematically as

1 Ag^0^ → Ag^+^ + e^−^

followed by oxygen reduction reactions such as

2 O_2_ + e^−^ → ˙O_2_^−^

which contribute to reactive oxygen species generation and subsequent cellular damage. These reactions underscore a central point: the biological activity of Ag nanoparticles in polymer matrices is inseparable from matrix controlled interfacial transport and from the structural stability of the nanoparticle phase.

#### 2.1.3 Photothermal theranostics and scale-resolved characterization

Photothermal therapy relies on the conversion of absorbed optical energy into localized heat and therefore demands a scale resolved understanding of absorption, thermal dissipation, and biological heat delivery. Yu *et al.*^44^ reviewed the development of noble metal nanoparticle based photothermal therapy and showed that therapeutic efficacy depends on both the efficiency of optical to thermal conversion and the spatial distribution of thermal dose within tissue. In polymer nanocomposites, one class of structural variables controls optical absorption and local field enhancement, whereas another governs thermal transport, interfacial heat resistance, and the redistribution of generated heat through the surrounding matrix. The observed temperature time profile is therefore a product of coupled optical and thermal descriptors rather than of nanoparticle composition alone. Adhikari *et al.*^45^ reviewed photothermal microscopy and demonstrated that absorption and energy dissipation may be resolved at the level of single nanoparticles. This experimental basis is particularly important because it discourages interpretation based solely on bulk temperature increase and instead supports a direct connection between nanoscale absorption processes and macroscale thermal response. Photothermal functionality in polymer nanocomposites should accordingly be analyzed as a multiscale phenomenon controlled by the interplay of plasmonic coupling, matrix constrained heat transfer, and the heterogeneity of thermal transport pathways.

A frequently used expression for photothermal conversion efficiency is
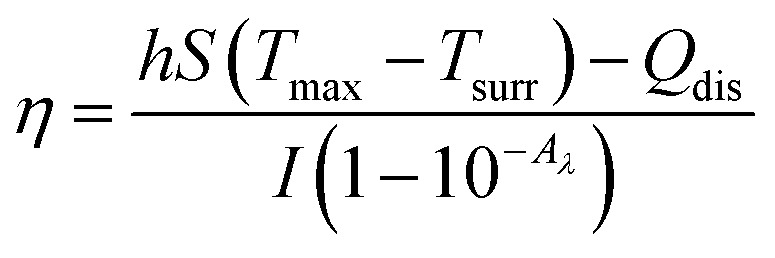
where, *hS* represents the heat transfer coefficient multiplied by the effective surface area, *T*_max_ is the equilibrium temperature under irradiation, *T*_surr_ is the ambient temperature, *Q*_dis_ denotes heat dissipation from the background system, *I* is the incident light power, and *A*_*λ*_ is the absorbance at the irradiation wavelength. In polymer nanocomposites, this parameter is strongly dependent on nanoparticle arrangement, matrix thermal conductivity, and the interfacial resistance to heat flow.

#### 2.1.4 Surface reactivity and application constraints of platinum and palladium nanoparticles

Platinum and palladium nanoparticles contribute multifunctionality primarily through surface mediated reactivity, catalytic activity, and catalysis related biomedical functions. Their practical deployment, however, is constrained by surface state stability, possible toxicity, and long term structural integrity. Jeyaraj *et al.*^46^ reviewed the synthesis, characterization, and biomedical applications of platinum nanoparticles and showed that reactivity and bioeffects are highly sensitive to particle size, morphology, and surface chemical condition. Variations in precursor chemistry, reducing environment, and post synthesis treatment may alter exposed crystal facets, surface ligands, and residual adsorbates, thereby producing substantial divergence in performance even within ostensibly similar systems. Phan *et al.*^47^ summarized the biomedical applications of palladium nanoparticles and stressed that utility requires simultaneous control over interfacial reactivity and biological safety. Joudeh *et al.*^48^ further reviewed palladium nanoparticle synthesis and applications and demonstrated that dispersion stability and surface state are highly dependent on preparation route. In polymer nanocomposites, these findings imply that Pt and Pd nanoparticles cannot be treated merely as nominal catalytic fillers. Their functionality is conditioned by surface accessibility, ligand environment, matrix imposed mass transport, and the capacity of the composite to preserve an active interface over time. Surface state control and interfacial regulation are therefore central to any meaningful discussion of Pt and Pd based polymer nanocomposites.

#### 2.1.5 Structural viewpoint on polymer–noble–metal nanocomposites

Folarin *et al.*^49^ reviewed polymer noble metal nanocomposites and emphasized the decisive role of polymer matrices in governing nanoparticle dispersion, interfacial bonding, and structural stability. This conclusion carries broad significance for the present field. The function associated with a particular metal species can only be expressed effectively when the surrounding polymer matrix generates an appropriate structural environment and maintains that environment under use conditions. Metal type determines the potential range of physical and chemical functionality. Composite microstructure determines whether that potential is translated into measurable performance with sufficient accessibility, reproducibility, and durability (Table 1).

**Table 1:** Functional roles, structural sensitivities, and application constraints of representative metal nanoparticles in polymer nanocomposites

Metal nanoparticles	Principal roles	Key structural sensitivities	Main constraints	References
Au NPs	Plasmonic response, photothermal conversion, biosensing	Particle size, morphology, interparticle gap, dielectric environment	High cost, aggregation induced signal broadening	33–35,40, 44, 45 and 49
Ag NPs	Antimicrobial activity, conductive transport	Surface oxidation, ion release, dispersion stability, aggregation state	Cytotoxicity risk, oxidation induced drift	41–43, 49 and 50–56
Pt NPs	Surface reactivity, catalytic and biomedical functions	Surface state, exposed facets, ligand residues	High cost, surface deactivation	46 and 49
Pd NPs	Catalytic activity, interface related response	Surface chemistry, dispersion stability, synthesis dependent structure	Toxicity concerns, reproducibility issues	47–49
Hybrid noble metal NPs	Synergistic multifunctionality	Composition ratio, nanoscale heterogeneity, structural stability	Complex synthesis and interpretation	33 and 49

### 2.2 Polymer matrices and architectures

Polymer matrices are not passive supporting media. They act as structure generating and function regulating phases that determine nanoparticle localization, confinement, migration, and long term stability through their chain dynamics, phase behavior, free volume distribution, and network architecture. Fu *et al.*^57^ discussed structural hierarchy, interfacial effects, and property response in polymer nanocomposites and argued that effective performance must be interpreted from measurable structural and interfacial levels rather than from nominal composition alone. Hiremath *et al.*^58^ further reviewed the processing and application of nanoparticle filled polymer nanocomposites and showed that matrix selection, processing route, dispersion quality, and stability are inseparably linked. Gobena and Woldeyonnes^59^ summarized synthesis methods and characterization strategies and made clear that different polymer systems privilege different structural variables and consequently require different criteria for valid comparison.

**Fig. 2:**
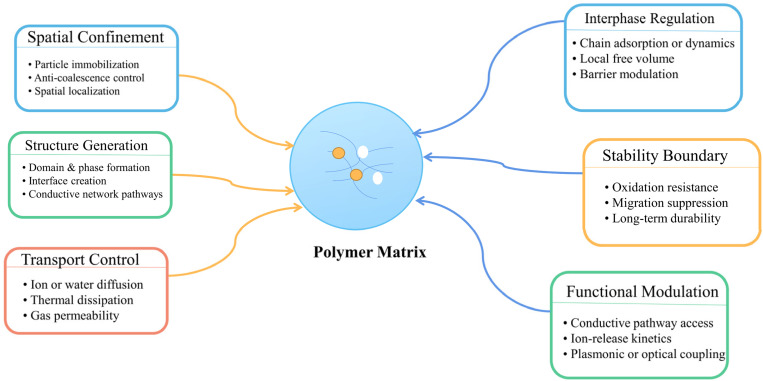
Roles of polymer matrices as structure generators, transport regulators, and stability boundaries in metal nanoparticle based nanocomposites.

Under multifunctional requirements, matrix selection is restricted not only by processability, but also by the manner in which different target functions must coexist. Musa *et al.*^60^ reviewed recent progress and challenges in nano enhanced polymer composites and concluded that multifunctional integration often narrows the processing window, intensifies environmental and health concerns, and undermines long term stability. These issues become even more consequential in metal nanoparticle systems, where oxidation, migration, interfacial aging, and local reorganization of the metallic phase may alter conductive pathways, release kinetics, and field response over time. The polymer matrix therefore establishes not only a structural template, but also the boundary conditions within which multifunctionality remains stable. Ma *et al.*,^61^ using machine learning assisted analysis of composition and property relationships in NanoMine, further demonstrated that unified structural parameterization is essential for meaningful comparison across systems and for the development of predictive design strategies. From a theoretical perspective, the role of the polymer matrix can be summarized in three respects. First, it provides structural degrees of freedom through chain mobility, confinement, and phase organization. Second, it defines the stability limits of the composite through swelling behavior, diffusion control, and resistance to structural drift. Third, it regulates the accessibility of functional interfaces and transport pathways, thereby conditioning electrical, optical, thermal, catalytic, and biological performance (Table 2 and Fig. 2).

**Table 2:** Structural roles of polymer matrices and their implications for multifunctional nanocomposite design

Matrix role	Structural effect	Functional implication	References
Spatial confinement	Restrains migration and aggregation of nanoparticles	Improves structural stability and dispersion uniformity	57–59
Structure generation	Creates domains, interfaces, and network pathways	Enables controlled conductive, optical, and biological functions	57, 58 and 61
Interfacial regulation	Modifies interphase thickness and local free volume	Governs charge transport, plasmonic coupling, and release behavior	57, 60 and 61
Transport control	Regulates diffusion, swelling, and thermal dissipation	Affects antimicrobial activity, photothermal response, and biosafety	60 and 50–56
Stability boundary	Limits oxidation, migration, and structural drift	Determines long term durability and device reliability	60 and 54–56

### 2.3 Interphase, dispersion, and percolation behavior

The concept of interphase is indispensable for understanding why the effective structure of a polymer nanocomposite differs fundamentally from its purely geometric description. Huang *et al.*^62^ reviewed interphase phenomena in polymer nanocomposites and concluded that interfacial regions possess chain conformation and dynamic behavior distinct from those of the bulk polymer, while contributing appreciably to effective volume fraction and macroscopic performance. This insight is especially important for metal nanoparticle systems because conductivity, photothermal response, and interfacial chemical activity are highly sensitive to interparticle separation and to the energetic barrier imposed by the surrounding matrix. Interphase thickness, local chemistry, and chain dynamics therefore influence effective tunneling distance, contact resistance, and functional accessibility.

Dispersion state and interfacial condition together determine how networks form and how transport proceeds. Chen *et al.*,^63^ in their review of carbon nanotube polymer nanocomposites, showed that interfacial interactions govern both load transfer and the stability of conductive pathways. Although their analysis concerned carbon based fillers, the underlying principle is directly applicable to metal nanoparticle systems. In such systems, interfacial bonding strength and interphase properties should be treated as explicit structural variables rather than as secondary background information. Zare and Rhee^64^ proposed conductivity calculations incorporating interphase assumptions and demonstrated that interphase thickness and interphase property strongly affect effective conductive volume fraction and apparent percolation behavior. Sharifzadeh *et al.*^65^ further analyzed the effects of aggregation and dispersion quality on electrical response and showed that poorly dispersed and highly aggregated systems differ from well dispersed systems not merely in connectivity, but also in the degree to which interphase effects and long term stability can be maintained. Poor dispersion introduces local heterogeneity, weakens reproducibility, and increases the likelihood of functional drift during service.

Percolation theory provides the fundamental theoretical basis for conductive and shielding behavior in these composites. Bauhofer and Kovacs^66^ analyzed electrical percolation in carbon nanotube polymer composites and showed that the percolation threshold and the associated critical behavior are governed by filler morphology, orientation, dispersion, and interfacial effects. In metal nanoparticle systems, similar logic applies, but the conductive network generally involves both direct contact conduction and tunneling mediated transport between closely spaced particles. Nan *et al.*^67^ reviewed the formation of effective conductive pathways and the development of electromagnetic shielding materials, emphasizing that shielding performance depends not only on elevated conductivity but also on multiscale conductive pathways and interfacial polarization loss. Zare *et al.*^68^ further demonstrated that neglect of deficient interphases in conductivity models leads to systematic deviation from observed behavior. The broader implication is clear: interphase design and dispersion control are essential to any predictive structure to property relationship in metal nanoparticle based polymer nanocomposites.

**Fig. 3:**
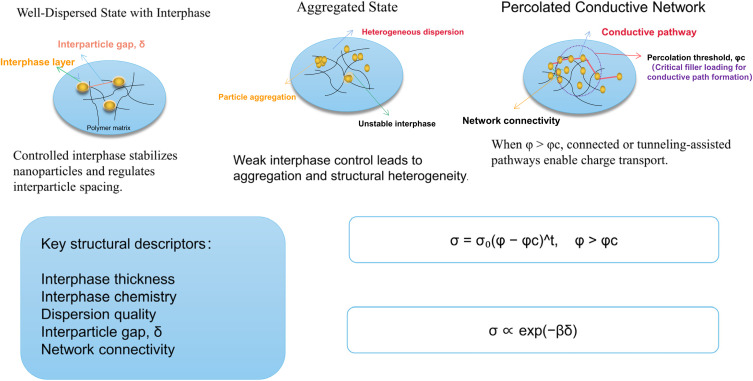
Interphase governed dispersion, network formation, and percolation behavior in metal nanoparticle based polymer nanocomposites.

The conductivity above the percolation threshold is commonly expressed as*σ* = *σ*_0_(*ϕ* − *ϕ*_c_)^*t*^, *ϕ* > *ϕ*_c_where, *σ* is electrical conductivity, *σ*_0_ is a scaling constant, *ϕ* is the filler volume fraction, *ϕ*_c_ is the percolation threshold, and *t* is the critical exponent. In metal nanoparticle systems, however, this expression must be read in conjunction with barrier controlled tunneling conduction, which may be approximated by*σ*_*t*_ ∝ exp(−*βg*)where, *g* denotes the effective interparticle gap and *β* characterizes attenuation through the interfacial barrier. These relations show that conductive behavior is determined not simply by how much metallic material is present, but by how the composite architecture regulates connectivity, spacing, and barrier controlled transport (Table 3 and Fig. 3).

**Table 3:** Structural descriptors of interphase, dispersion, and percolation governing transport and functional response

Structural descriptor	Physical meaning	Main affected functions	References
Interphase thickness	Functional region surrounding nanoparticles	Conductivity, shielding, photothermal dissipation	62, 64 and 68
Interphase chemistry	Local chemical and bonding state at the interface	Charge transport, bioactivity, catalytic stability	62–64 and 68
Dispersion quality	Uniformity of nanoparticle distribution	Conductive stability, sensing fidelity, optical uniformity	63, 65 and 66
Interparticle gap	Effective spacing between adjacent nanoparticles	Tunneling conduction, plasmonic coupling	62, 64 and 66
Network topology	Connectivity and continuity of functional pathways	Conductivity, EMI shielding, strain response	66, 67, 69 and 70
Percolation threshold	Critical filler fraction for continuous transport	Electrical transport and shielding transition	64 and 66–68

### 2.4 Definitions and metrics of multifunctionality

Multifunctionality in metal nanoparticle based polymer nanocomposites cannot be reduced to the coexistence of several isolated properties. It requires operational definition, metric consistency, and evaluation under conditions relevant to actual use. Nan *et al.*^69^ reviewed flexible nanocomposite conductors for electromagnetic interference shielding and showed that multifunctional performance must be assessed not only by electrical conductivity or shielding effectiveness under static conditions, but also by the ability of the material to preserve functional pathways under mechanical deformation. Li *et al.*^70^ further reviewed principles, fabrication methods, and applications of polymer based shielding composites and emphasized the importance of normalized reporting with respect to thickness and density. Without such normalization, apparent performance enhancement may merely reflect increased sample thickness or filler loading rather than genuine structural optimization.

In soft and biomedical materials, the problem becomes more complex because several functions are often required to operate simultaneously and under dynamic physiological conditions. Qiao *et al.*^50^ reported conductive self healing antibacterial hydrogel dressings and clarified the role of dual dynamic bonds in coupling repair behavior with antimicrobial function. Liu *et al.*^51^ developed self healing antibacterial conductive double network hydrogels for strain sensing and demonstrated that network architecture governs both mechanical toughness and electrical stability. Fang *et al.*^52^ discussed conductive hydrogels as intelligent dressings for chronic wound monitoring and healing and highlighted the need to link sensing performance with biological outcome. He *et al.*^53^ designed photothermal antibacterial antioxidant conductive self healing hydrogels with nitric oxide release and showed that energy conversion, antibacterial efficacy, and tissue repair relevant response must be evaluated together. Hu *et al.*^54^ reviewed multifunctional antibacterial hydrogels for chronic wound management and emphasized that long term biosafety and structural stability are as important as short term therapeutic effectiveness. Wang *et al.*^55^ summarized metal nanoparticle hybrid hydrogels and identified hard nanoparticle nodes and soft polymer networks as a structurally explicit basis for multifunctional design. Stevanović *et al.*^56^ reviewed nanomaterial integrated hydrogels for sustained drug delivery and likewise stressed the necessity of metric systems that connect material characterization with biomedical outcome.

These studies suggest that multifunctionality should be treated as a constrained multiobjective domain rather than as a collection of independent maxima. Electrical performance must be interpreted together with mechanical durability and cyclic retention. Photothermal performance must be evaluated together with antibacterial efficacy and biocompatibility. Electromagnetic shielding must be reported together with thickness normalized or density normalized descriptors and with functional retention under repeated deformation. The appropriate question is therefore not which single property reaches the highest value, but which structural configuration yields the most balanced and reliable combination of functions under application specific constraints.

**Fig. 4:**
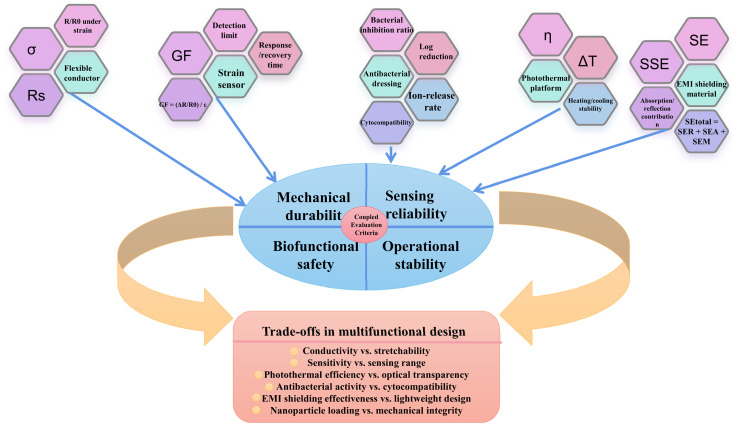
Multifunctional performance metrics and coupled evaluation criteria for metal nanoparticle–polymer nanocomposites.

For strain responsive conductive systems, the gauge factor is commonly defined as
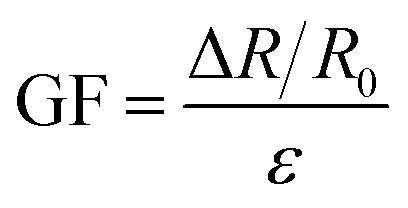
where, Δ*R*/*R*_0_ is the relative change in resistance and *ε* is the applied strain. For electromagnetic shielding systems, the total shielding effectiveness is expressed asSE_T_ = SE_R_ + SE_A_ + SE_M_where, SE_R_, SE_A_, and SE_M_ denote the contributions from reflection, absorption, and multiple internal reflections, respectively. These metrics further illustrate that multifunctionality must be assessed through integrated descriptor and performance relationships, not through isolated endpoint values (Table 4 and Fig. 4).

**Table 4:** Representative multifunctionality metrics and corresponding structural evaluation logic

Functional domain	Core metrics	Coupled metrics required	References
Electrical transport	Conductivity, sheet resistance	Strain retention, cyclic stability	50–52,69,70
Strain sensing	Gauge factor, response stability	Strain range, hysteresis, fatigue resistance	51–53
Photothermal response	Photothermal conversion efficiency, temperature rise	Irradiation stability, biocompatibility, antibacterial effect	44, 45 and 53–55
Antimicrobial function	Inhibition ratio, log reduction	Ion release profile, cytocompatibility, durability	41–43, 50 and 53–56
EMI shielding	Total shielding effectiveness	Thickness normalized performance, deformation retention	67, 69 and 70
Self healing soft systems	Healing efficiency, conductivity recovery	Mechanical recovery, functional retention	50–55

## 3 Controllable fabrication strategies for multifunctional metal nanoparticle based polymer nanocomposites

For multifunctional metal nanoparticle-based polymer nanocomposites, fabrication strategy is not merely a processing option, but the primary means by which structural descriptors are established and subsequently translated into functional performance. From a materials perspective, controllable fabrication seeks to define reproducible links among process variables, nanoparticle organization, interfacial states, and device level outputs, so that particle size, spatial distribution, interphase structure, and network connectivity are regulated rather than incidentally obtained. The central question is therefore not simply whether metal nanoparticles can be incorporated into polymer matrices, but whether their formation, localization, and evolution can be directed with sufficient reproducibility for application specific requirements.

**Fig. 5:**
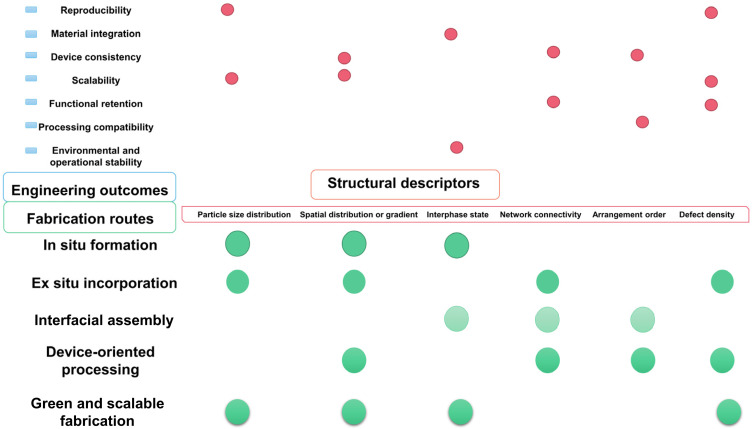
Relationship among fabrication routes, structural descriptors, and engineering outcomes in metal nanoparticle–polymer nanocomposites.

Current studies can be grouped into several major fabrication paradigms. *In situ* formation exploits the constraint field of the polymer matrix to regulate nucleation, growth, and coarsening, often improving interfacial integration and suppressing secondary aggregation. *Ex situ* incorporation decouples nanoparticle synthesis from polymer shaping, thereby expanding formulation flexibility but shifting the control problem toward dispersion preservation and interfacial accessibility during casting and curing. Interfacial assembly and ordered structuring reduce configurational randomness by organizing nanoparticles into more deterministic architectures, which is particularly valuable where optical, electrical, or sensing responses demand narrow structural tolerances. Device oriented processing introduces geometric design as a further control variable through printing, meshes, fibrous networks, and additive manufacturing. Green and scalable routes have also received growing attention, although their principal challenge remains the maintenance of descriptor stability and batch consistency under practical manufacturing conditions (Fig. 5).

### 3.1 *In situ* formation of metal nanoparticles within polymer matrices

#### 3.1.1 State of the art and mechanistic basis

Among the available routes, *in situ* formation is distinguished by the simultaneous coupling of nanoparticle generation and matrix evolution. Its main advantage is that nucleation and growth proceed within a chemically and physically constrained environment, so that the polymer phase acts as an active regulator of particle formation rather than as a passive host. Functional groups, chain adsorption, diffusion restriction, and local viscoelastic confinement jointly reshape nucleation and growth kinetics, often reducing uncontrolled migration and coalescence and thereby improving the reproducibility of size and spatial distributions.^71–76^

Across thin films, crosslinked networks, and confined soft domains, the literature converges on a common conclusion: the value of *in situ* routes lies not only in nanoparticle generation itself, but in their ability to coordinate particle formation, interfacial adhesion, and spatial confinement within a single process window.^71–74^ Recent work has further shifted from controlling mean particle size alone to regulating distribution width, spatial gradients, and interphase states, which are more directly relevant to conductive continuity, photothermal localization, and controlled antimicrobial release.^74–77^ The main unresolved issue concerns scale translation, since nonuniform transport, solvent removal, or field intensity can broaden structural distributions and introduce spatial heterogeneity even when nominal chemistry remains unchanged.^74,75^

#### 3.1.2 Evidence base and representative systems

To evaluate the practical maturity of *in situ* formation, the most relevant evidence is whether specific process variables can be consistently mapped onto structural outcomes across different polymer platforms. Representative studies therefore need to be read not only as isolated examples, but as evidence that the route has evolved from empirical synthesis toward mechanism-guided controllability. Adnan *et al.*^71^ synthesized a comprehensive framework for *in situ* synthesis of hybrid inorganic polymer nanocomposites, reporting that *in situ* routes frequently yield strong interfacial adhesion and reduced secondary aggregation because inorganic phase formation and polymer consolidation occur within one coupled environment. Fujioka *et al.*^72^ prepared Ni nanoparticles by liquid-phase reduction within polyimide systems and fabricated metal nanoparticle polyimide composite films, illustrating how restricted diffusion after consolidation may suppress coarsening and spatial drift, stabilizing structure descriptors over time. Lee *et al.*^73^ demonstrated soft plasma electrochemical reduction within metallo supramolecular polymer films, where potential and charge injection served as external control variables governing reduction rates and spatial occurrence, enabling location-controlled distributions. Ramesh *et al.*^74^ emphasized that film formation introduces local concentration changes, diffusion gradients, and chain adsorption effects, leading to thickness-dependent distributions, so film thickness and evaporation history function as structural control variables rather than incidental conditions. *In situ* literature offers strong evidence linking process variables to interphase formation and distribution outcomes. Remaining gaps are dominated by quantitative mapping from process histories to distribution statistics under scaled reactors and large-area films.

#### 3.1.3 Chemical and photochemical reduction routes

##### 3.1.3.1 Mechanistic framework and research characteristics

Within *in situ* fabrication, reduction chemistry defines the most direct pathway from precursor state to nanoparticle formation. From a mechanistic standpoint, controllability in these routes depends on whether electron supply, reaction locality, and stabilization dynamics can be synchronized so that nucleation density and growth trajectories remain programmable. This is why chemical and photochemical reduction are often treated as two related but distinct subfamilies: the former emphasizes chemical environment regulation, whereas the latter introduces spatial and temporal field control.

Chemical reduction is governed by competition between nucleation and growth rates, modulated by ligand environments and polymer functional groups that stabilize surfaces and regulate facet-selective adsorption. Photochemical reduction adds spatiotemporal programmability by controlling flux of photogenerated electrons or radicals through light intensity, wavelength, and exposure patterns, enabling localized nucleation and gradient architectures.^75–77^ Two research characteristics dominate recent progress. Chemical routes have increasingly adopted reduction–stabilization co-design, particularly in green systems where stabilization mechanisms directly regulate antimicrobial release and long-term structural integrity.^78^ Photochemical routes have evolved from nanoparticle formation toward deterministic patterning and stratified localization in microstructures, supporting device-compatible functional zoning.^75,76^

##### 3.1.3.2 Evidence base and representative systems

The representative systems in this category collectively illustrate how controllability shifts from purely chemical conversion efficiency to integrated regulation of structure, localization, and function. Their value lies in demonstrating that reduction routes can serve not only as synthesis tools but also as architectural design tools for multifunctional composites.

Bhangi and Ray^79^ synthesized Cu nanoparticles in chitosan-entrapped copolymer matrices and linked confinement and complexation to preserved accessible surface sites and suppressed aggregation, supporting photocatalysis and heavy-metal adsorption as structure-dependent functions. Sankar *et al.*^80^ studied copper alumina poly(aniline-*co*-indole) nanocomposites and highlighted sensitivity of transport pathways and interfacial barriers to particle scale and interphase states, which is critical for electrical and gas sensing mechanisms. Sharma *et al.*^78^ reviewed green synthesis and antimicrobial activity of silver nanoparticles, arguing that reaction conditions and stabilizers determine size and aggregation morphology, which in turn controls ion release and surface reactivity, establishing reproducible stability – release windows as central control targets. Sakamoto *et al.*^77^ summarized light-driven construction mechanisms and identified photon-controlled radical or electron flux as direct handle over nucleation density and growth dynamics. Nadal *et al.*^75^ demonstrated *in situ* Au nanoparticle formation under concentrated sunlight in polymer films, showing solar flux functions as continuous external control variable for size and shape distributions. Park *et al.*^76^ achieved Ag nanoparticle patterns in microstructures through photo-thermal reduction, where exposure patterns and boundary-defined transport conditions jointly determined formation sites and particle densities, enabling spatially defined functional zones. Chemical reduction is mature in mechanistic vocabulary and platform diversity, while green routes still require stronger control over residuals and release kinetics variability. Photochemical routes exhibit high spatial controllability, yet scaling patterned features to large areas remains constrained by optical field uniformity and heat transport control.

#### 3.1.4 Template assisted and microgel mediated *in situ* synthesis

If reduction routes primarily control reaction kinetics, template-assisted and microgel-mediated strategies primarily control reaction topology. Their theoretical significance lies in converting random nucleation events into site-defined events by prebuilding soft domains, pores, or crosslinked regions that prescribe where ions accumulate and where particles can grow. In this framework, the template is not only a support medium but a mesoscale regulator of spatial probability distribution.

Template and gel mediated strategies explicitly define nucleation locations *via* preorganized soft domains. Crosslink density, pore size, functional-site distribution, and swelling behavior modulate local ion enrichment, diffusion pathways, and supersaturation, thereby controlling nucleation site density, upper bounds of particle size, and aggregation scale. The evidence base is strong in bio-based networks and gels, where high site density and tunable channels provide robust confinement and anchoring.^81–83^ Application-oriented studies increasingly emphasize interfacial localization. Antibacterial and disinfection systems favor enrichment near accessible interfaces to maximize contact efficiency, whereas magnetic gels prioritize channel accessibility for mass transport and recoverability.

The representative studies in this area show that confinement is most valuable when it is translated into application-relevant localization rather than only smaller particle size. Accordingly, the literature increasingly evaluates templates by how effectively they organize accessibility, transport, and recovery pathways in addition to stabilizing nanoparticles.

Sriplai and Pinitsoontorn^81^ reviewed bacterial cellulose based magnetic nanocomposites and argued that continuous channels and hydroxyl-rich networks stabilize magnetic nanoparticles while retaining flexibility and magnetic responsiveness. Hajareh Haghighi *et al.*^82^ demonstrated peptide-based magnetogels where self-assembled network domains regulated enrichment and localization of metal species, shaping accessibility and transport that govern heavy-metal removal performance. Leudjo Taka *et al.*^83^ reviewed antimicrobial biopolymer inorganic nanoparticle composites for water disinfection and emphasized that antibacterial efficacy depends on interfacial accessibility and localization, supporting template-guided interfacial structuring as practical design direction. Confinement-based control is well established at laboratory scale and in bio-based platforms. Generalizable quantitative rules that link network descriptors to nanoparticle distribution statistics across diverse polymers remain comparatively limited.

#### 3.1.5 Kinetic control and reaction engineering constraints

No fabrication strategy can be considered truly controllable unless structural descriptors remain stable when reaction conditions are translated beyond laboratory ideality. For this reason, kinetic control must be interpreted together with reaction engineering constraints. The central theoretical issue is that local differences in concentration, energy input, or transport rate are amplified during early-stage nucleation and then frozen into final structure distributions, making process history an intrinsic part of material design.

Kinetic control is expressed through statistical structure descriptors rather than single-point values. Nucleation density sets particle number density and mean size, while growth, ripening, and coalescence determine distribution width and aggregation scale. Polymer diffusion constraints amplify early-stage differences and may induce spatial gradients.^74,77^ In externally driven systems, energy or charge injection rates become dominant parameters, and flux fluctuations can map directly to structural variability and performance drift.^73,75^ Although controllable structures are routinely achieved at laboratory scale, scale translation often introduces mixing and mass-transfer nonuniformity, producing broadened distributions, bimodality, or spatial heterogeneity. Time–temperature–mixing histories therefore need to be treated as controlled process variables under explicit quality control frameworks.^74,75^

The following studies are especially important because they reveal that variability often originates not from nominal chemistry, but from hidden heterogeneities in process history. They therefore shift the discussion of controllability from material formulation alone toward process-field management. Ramesh *et al.*^74^ highlighted strong coupling between film formation and diffusion gradients, where film thickness and solvent evaporation history modulate local concentration trajectories and thus nucleation and ripening pathways.^74^ Lee *et al.*^73^ showed that potential and transport limitations control spatial distributions of reduction, implying that uniform electrochemical conditions across large-area films are required for structural consistency.^73^ Nadal *et al.*^75^ indicated that solar flux governs nucleation–growth competition in sunlight-driven Au formation, so flux stability and exposure time control directly affect distribution reproducibility.^75^ Kinetic explanations are conceptually mature, but engineering-level translation into reactor-scale, large-area, and continuous processes remains principal gap, particularly for maintaining uniform supersaturation and uniform field conditions.^74,75^

### 3.2 *Ex situ* prepared nanoparticles incorporated into polymers

#### 3.2.1 State of the art and mechanistic basis

Compared with *in situ* formation, *ex situ* incorporation separates nanoparticle generation from matrix construction and thereby changes the main control problem. The theoretical advantage of this route lies in its modularity: particle size, composition, and morphology can be optimized independently before composite formation. However, once synthesis and shaping are decoupled, the dominant challenge shifts to preserving dispersion state and interfacial accessibility during solvent exchange, casting, and curing, where irreversible structural randomness can be introduced.

*Ex situ* routes are widely adopted in engineering contexts because nanoparticle synthesis and polymer shaping can be optimized independently. Strong foundations exist in sensing films, separation membranes, and photopolymer processing, where method sets are comparatively systematic. Two persistent challenges dominate. Solvent exchange and drying may induce ligand desorption and secondary aggregation, which reduces interfacial accessibility and introduces randomness. Curing or film formation locks structural states, so dispersion stability must be secured prior to curing-front development; post-processing rarely reconstructs controlled distributions once defects are frozen in.

#### 3.2.2 Evidence base and representative systems

The representative literature demonstrates that *ex situ* controllability depends less on nominal nanoparticle loading and more on whether the predesigned particle characteristics survive the shaping process. In this sense, the route is governed by preservation of accessibility and distribution integrity rather than synthesis precision alone. Qiao *et al.*^84^ fabricated zwitterionic copolymer membranes for oil–water separation, demonstrating decisive influence of solution casting on morphology and interfacial properties, which supports viewing evaporation history as a control variable for nanoparticle migration and enrichment in composite membranes. Prakash *et al.*^85^ discussed polymer thin films embedded with metal nanoparticles for electrochemical biosensors, emphasizing dependence of responses on interfacial accessibility of metal surfaces and continuity of electron transport channels; loading alone is insufficient descriptor without accessibility metrics. Im *et al.*^86^ reviewed integration of metal nanoparticles with two-photon polymerization and stressed coupling among nanoparticle scattering absorption, curing dynamics, and microdefect generation, requiring co-optimization of formulation and processing to preserve resolution and functional continuity. *Ex situ* routes are mature in processing variety and application coverage. Structural randomness introduced by drying and curing remains dominant source of variability, and standardized metrics for interfacial accessibility are still underdeveloped relative to loading-based descriptions.

### 3.3 Interfacial assembly and ordered nanostructuring

#### 3.3.1 State of the art and mechanistic basis

When multifunctional performance is highly sensitive to interparticle spacing, orientation, or collective coupling, random dispersion becomes intrinsically insufficient. Interfacial assembly and ordered nano structuring address this issue by reducing configurational entropy and replacing stochastic distributions with designed architectures. The theoretical premise is that ordering transforms nanoparticle arrangement itself into a controllable variable, thereby improving spectral, electrical, and mechanical reproducibility.

Interfacial assembly is strongly motivated by strict requirements on structural consistency in sensing and photonics. Random dispersion often yields uncontrolled variations in aggregation scale and interparticle spacing, broadening plasmonic spectra and destabilizing transport pathways. Ordered architectures reduce configurational randomness and improve reproducibility and device consistency.^87,88^ Grafting and brush strategies further convert interphase into tunable structural element, bringing interparticle spacing and barrier states into design space, enabling tractable trade-offs among percolation thresholds, mechanical reinforcement, and optical coupling.^89,90^

#### 3.3.2 Evidence base and representative systems

The literature in this category is important because it shows that ordered structures do not merely enhance performance magnitude; more importantly, they suppress uncertainty in structure–property relationships. This feature is particularly valuable for devices requiring narrow tolerances and repeatable signal outputs. Abhishek *et al.*^87^ discussed metal conducting polymer hybrid composites for electrochemical sensing and highlighted that electron transfer depends on interfacial layer structure and exposure of active sites, both tunable through assembly protocols. Scroccarello *et al.*^88^ reviewed plasmonic sensing in solid supports and emphasized that structural consistency governs optical stability; ordered integration reduces spectral broadening driven by random aggregation. Cazotti *et al.*^89^ presented controlled graft modification methodology and clarified that graft density and chain length provide spacing control, which maps to tunneling distance and percolation thresholds. Olson *et al.*^90^ argued that entropy-driven self-assembly supports stable dispersion while forming hierarchical structures, offering mechanistic basis for programmable architectures under multifunctional constraints. Ordered structuring has strong conceptual and experimental basis for improving reproducibility. Industrially scalable assembly protocols with high throughput and broad substrate compatibility are still less established than laboratory-scale demonstrations.

### 3.4 Advanced processing and patterning for device-oriented architectures

#### 3.4.1 State of the art and mechanistic basis

At the device level, structural control is no longer limited to nanoscale particle placement but extends to mesoscale and macroscale geometry. Advanced processing and patterning are therefore theoretically significant because they add architectural freedom as a new degree of control. In such systems, functional reliability depends on how geometry redistributes strain, maintains conductive continuity, and localizes active regions under service conditions.

Device-oriented processing treats geometry as primary control variable for functional retention. In flexible and stretchable settings, functional stability frequently depends on maintaining conductive pathways under deformation, which is governed by geometry-driven redistribution of local strain. High-resolution printing and metallic meshes provide established foundations for transparent conductors and flexible optoelectronics, while fibrous networks provide transferable paradigms for self-monitoring and sensing. Additive manufacturing supplies route for embedding functional units into three-dimensional architectures.^86,91–96^

#### 3.4.2 Evidence base and representative systems

The representative studies collectively indicate that device-oriented fabrication succeeds when processing path, local curing behavior, and network continuity are designed in a coordinated manner. Thus, geometry should be understood not as a secondary packaging issue but as a primary determinant of multifunctional retention.

Im *et al.*^86^ emphasized coupling between nanoparticle presence, curing dynamics, and achievable resolution in two-photon polymerization, reflecting dependence on formulation and process windows.^86^ Zhang *et al.*^91^ demonstrated impact-resistant self-monitoring nanofibrous composites, showing that fibrous architectures can couple mechanical dissipation with signal outputs while preserving continuity.^91^ Meda *et al.*^92^ reviewed nano-engineered textiles and stressed that stable integration on large-area flexible substrates requires synergy between fibrous network design and surface modification.^92^ Kim *et al.*^93^ analyzed micrometer-scale copper electrode printing and showed that ink formulation and process optimization determine continuity and resistance, which govern device consistency.^93^ Zhang *et al.*^94^ reviewed metallic meshes and highlighted geometric control over transparency, conductivity, and mechanical compliance.^94^ Han *et al.*^95^ linked percolated network descriptors to sensing mechanism and cyclic stability in transparent conductive nanocellulose nanocomposites.^95^ Rayhan *et al.*^96^ summarized additive manufacturing of nanocomposites and argued that process parameter windows govern structural continuity and functional consistency in printed parts.^96^ Device-oriented processing is mature in proof-of-concept diversity and geometry-enabled design rules. Predictive coupling models that integrate printing path, curing history, and nanoparticle network evolution remain insufficient for robust parameter transfer across equipment and scales.^86,96^

#### 3.4.3 Advanced manufacturing routes for device-oriented polymer/metal nanoparticle nanocomposites

The fabrication of metal nanoparticle/polymer nanocomposites has gradually moved beyond conventional coating, casting, and solution blending toward more device-oriented routes, including additive manufacturing, melt processing, vapor-phase deposition, sol–gel treatment, and electrospinning. These methods are important not simply because they offer new processing options, but because they allow better control over device geometry, particle distribution, interfacial structure, and functional continuity.

Additive manufacturing is useful for producing complex three-dimensional structures with spatially programmable functions. Extrusion-based printing is generally suitable for thermoplastic filaments containing metal nanoparticles, whereas vat photopolymerization is more appropriate for high-resolution photocurable systems. Cu nanoparticle-filled photopolymer resins have been processed by vat photopolymerization, and *in situ* photoreduction has been used to generate Ag nanoparticles during printing to form conductive hybrid structures.^97,98^ However, these methods are sensitive to formulation and processing conditions. Particle sedimentation, light scattering, curing inhibition, and discontinuous conductive pathways can reduce printing quality and reproducibility.

Melt processing is more suitable for scalable production, especially extrusion and filament fabrication. Reactive melt mixing has been used to prepare PLA/Ag nanocomposite filaments, where *in situ* Ag nanoparticle formation helps reduce the agglomeration often observed when preformed nanoparticles are directly compounded.^99^ Related studies also show that particle dispersion, processing temperature, mixing/extrusion history, and thermally induced structural evolution are critical for melt-processed metal nanoparticle/polymer nanocomposites.^100,101^ Although this route offers high throughput and good industrial compatibility, it may also cause polymer degradation, metal nanoparticle oxidation or coalescence, and loss of nanoscale dispersion after repeated thermal cycling.

Vapor-phase deposition offers a dry route for preparing polymer–metal nanocomposite thin films, especially for coating-type electrical and sensing applications.^102–104^ Co-sputtering can disperse metal nanoclusters within a polymer matrix, and the resulting electrical behavior strongly depends on metal loading and percolation state. Near the percolation threshold, changes in intercluster spacing can produce measurable responses to volatile organic compounds, making these films useful for gas sensing.^105^ However, this route is mainly limited to thin-film architectures and is constrained by vacuum processing, deposition-parameter sensitivity, possible polymer damage, and interfacial stability issues.

Sol–gel processing plays a different role and is more commonly used for metal oxide/polymer nanocomposites than for elemental metal nanoparticle systems. It can form inorganic nanodomains and organic–inorganic interphases that improve dispersion and regulate interfacial transport. PP/TiO_2_ and TiO_2_/polymer systems, for example, have shown antibacterial or photocatalytic performance, although poor control of hydrolysis and condensation may lead to phase separation, brittleness, or reduced matrix toughness.^106,107^

Electrospinning is well suited for flexible membranes, wearable substrates, filtration media, and lightweight EMI shielding structures. It produces continuous nanofibrous networks with high surface area, which facilitates later metallization or nanoparticle decoration. Electrospun polymer nanofiber membranes incorporating Ag nanowires, Ni particles, or other metallic components have shown good EMI shielding performance, indicating that metal-modified fiber surfaces can form conductive pathways while maintaining low density, flexibility, and thermal stability.^108,109^ In these systems, fiber orientation, porosity, nanoparticle loading mode, and inter-fiber contact are key factors controlling functional stability.

Overall, advanced manufacturing routes should be evaluated by their ability to control device geometry, nanoparticle dispersion, interfacial stability, and pathway continuity. Parameters such as printing fidelity, melt stability, deposition adhesion, porosity, and conductivity retention under deformation are often more meaningful than filler loading or process name alone.

### 3.5 Green and scalable fabrication approaches

From the perspective of industrial translation, fabrication cannot be considered advanced unless controllability is maintained under environmentally benign and scalable conditions. Green and scalable fabrication therefore introduces a stricter criterion than laboratory proof-of-concept: it requires simultaneous control over reaction efficiency, interfacial cleanliness, residual chemistry, and batch-level reproducibility. In other words, this category evaluates whether sustainability can be achieved without sacrificing descriptor stability.

Green fabrication and scalable manufacturing are expanding, yet maturity remains lower than that of *in situ*, *ex situ*, and assembly routes. Green routes must achieve controllable nucleation and stable dispersion under mild conditions while minimizing residuals that alter interphase chemistry and release pathways. Sharma *et al.*^78^ showed that green Ag systems can exhibit distinct stabilization mechanisms that shape size, aggregation, ion release, and antimicrobial effects, implying that stability and reproducible release kinetics windows represent core evaluation criteria.^78^ Sabín López *et al.*^110^ demonstrated aqueous synthesis of magnetic green nanoparticle molecularly imprinted polymers, supporting feasibility of functional materials under mild conditions.^110^ For industrial translation, Sammasagi *et al.*^111^ summarized scale-up and quality control challenges in nanoformulation manufacturing, identifying batch consistency and process monitoring as central bottlenecks. In metal nanoparticle polymer composites, this corresponds to drift in size distribution, enlarged aggregation scales, and residual-induced interfacial destabilization, which demand conversion of key structural descriptors into monitored process indicators and standardized characterization protocols.^111^ Green and scalable routes are active and application-driven, but robust quality-control frameworks and standardized descriptors remain less mature than for conventional routes, particularly when multifunctionality imposes tight windows on release and stability.^78,111^

#### 3.5.1 Scale-induced structural heterogeneity during scalable fabrication

Scale-up introduces a structural problem that is often less visible in laboratory-scale demonstrations: scale-induced heterogeneity. Metal nanoparticle–polymer nanocomposites that appear uniform in small specimens may develop pronounced spatial variations during large-area coating, roll-to-roll processing, melt extrusion, electrospinning, bulk curing, or additive manufacturing. These variations originate from gradients in temperature, solvent evaporation, shear history, residence time, precursor concentration, light intensity, curing-front velocity, and local viscosity. Because nanoparticle nucleation, growth, migration, aggregation, and network formation are highly sensitive to these local process fields, scale-up can transform a nominally identical formulation into a spatially heterogeneous architecture with nonuniform particle size, concentration, interparticle spacing, interphase structure, and network connectivity.

This issue is especially important for multifunctional metal nanoparticle–polymer nanocomposites, whose performance is rarely governed by average metal loading alone. Conductive systems can cross the percolation threshold locally but remain poorly connected elsewhere, leading to heterogeneous conductivity and unstable current pathways. Plasmonic and photothermal systems may suffer from uneven aggregation, spectral broadening, and localized overheating. Antibacterial and catalytic composites can exhibit nonuniform metal exposure, causing spatially inconsistent ion release or active-site accessibility. Electromagnetic shielding materials are likewise sensitive to thickness variation, pore gradients, and discontinuous conductive pathways, all of which can decouple nominal composition from device-level performance.

The form of heterogeneity depends on the processing route. *In situ* synthesis may generate position-dependent nucleation because of nonuniform diffusion, reduction, or irradiation. *Ex situ* incorporation can be affected by sedimentation, solvent removal, curing shrinkage, and filler migration before matrix consolidation. Melt processing introduces shear and thermal histories that may alter dispersion or accelerate particle coalescence. Additive manufacturing can create anisotropic distributions through nozzle flow, optical attenuation, curing-depth variation, and layer-by-layer accumulation. Electrospinning and coating further introduce heterogeneity through evaporation kinetics, deposition uniformity, fiber packing, and junction-density variations.

Accordingly, scalable fabrication should be assessed through spatially resolved descriptors rather than average filler content. Particle-size distribution, aggregation state, through-thickness concentration profile, lateral uniformity, interparticle spacing, porosity, metal surface exposure, interfacial chemical state, and local electrical or thermal conductivity are more informative than nominal composition alone. The same average loading may correspond to a uniform but sub-percolated dispersion, a continuous functional network, or a heterogeneous structure containing both conductive clusters and inactive regions.

Mitigating scale-induced heterogeneity requires process windows that preserve descriptor stability during manufacturing. Spatially resolved quality control, including electrical mapping, thermal imaging, optical inspection, rheological monitoring, spectroscopy, and image-based microstructure analysis, can help track structural drift during scale-up. Ultimately, scalable fabrication should be treated as a process–structure–property challenge rather than a simple increase in production volume. Practical translation will depend on whether nanoscale dispersion, interphase accessibility, and mesoscale network continuity can be maintained across large-area, continuous, and batch-to-batch processing.

### 3.6 Comparative analysis and process–structure maps

A comparative framework is necessary because no single fabrication route simultaneously optimizes all structural descriptors and engineering requirements. The purpose of process–structure mapping is therefore to identify which route offers the highest controllability for a given target function, and under which constraints its advantages begin to weaken. Such mapping is also important for shifting the discussion from isolated methods to a unified design logic based on descriptor-oriented selection.

Comparative evaluation requires joint expression of structural degrees of freedom and engineering constraints. *In situ* routes excel in interfacial adhesion, spatial gradients, and suppression of secondary aggregation, as represented by Ni polyimide composite films and electrochemical *in situ* formation in polymer films.^72,73^*Ex situ* routes excel in predefining size and shape and enabling flexible shaping windows, as represented by membranes, biosensor films, and two-photon polymerization systems.^84–86^ Ordered structuring excels in consistency and reproducibility, as represented by electrochemical sensing platforms and solid-supported plasmonic structures, while grafting and self-assembly introduce spacing and barrier-state control.^87–90^ Device-oriented processing leverages geometry to achieve designed synergy between conductive pathways and strain distributions, as represented by micrometer electrode printing, metallic mesh optoelectronics, and fibrous sensing architectures.^91–95^ Green and scalable routes require process monitoring and quality-control frameworks and remain dominated by method exploration and application validation at present stage.^78,110,111^ Accordingly, process–structure mapping can be formulated by linking each route to observable structural descriptors and explicit engineering limits. Observable descriptors include width of size distribution, fraction of anisotropic morphologies, magnitude of spatial gradients, network connectivity, effective interphase thickness and chemical states, degree of ordering, and defect density. Engineering limits include uniformity of solar flux or electrochemical potential, mixing and mass-transfer conditions, solvent evaporation history, curing-front stability, shear and temperature fields, and capacity for batch-consistent quality control. Comparative maps are increasingly feasible because descriptor sets are converging across subfields. Lack of unified reporting standards for accessibility, interphase chemistry, and long-term drift still limits cross-paper meta-analysis and quantitative synthesis.

## 4 Structure–property relationships governing multifunctional performance in metal nanoparticle–polymer nanocomposites

Structure–property relationships provide the central framework for understanding multifunctional metal nanoparticle–polymer nanocomposites. In these systems, macroscopic performance is not determined by composition alone, but by the collective effects of particle morphology, spatial distribution, interfacial organization, network topology, and their evolution under service conditions. Accordingly, controllable fabrication is meaningful only when processing variables can be translated into reproducible structural descriptors that govern transport, optical coupling, mechanical reinforcement, and environmental stability. This section therefore examines how morphology and dispersion, interphase-mediated networking, multifunctional coupling, multiscale analysis, and long-term durability jointly define performance in these materials.

### 4.1 Morphological regulation of electrical, dielectric, optical, and mechanical responses

Morphology and dispersion are the most immediate structural variables controlling multifunctional response. Even at similar filler fractions, changes in clustering, aspect ratio, orientation, and interparticle spacing can shift the dominant transport pathway and alter electrical or dielectric performance by orders of magnitude. Recent studies on metallic nanoparticle films, silver nanowire networks, and aligned nickel nanowire composites consistently show that conductivity and dielectric response are determined primarily by local connectivity, tunneling distance, and anisotropic topology rather than by nominal loading alone.^112–114^ Electrical performance in these materials is therefore better interpreted as a topology governed outcome than as a direct consequence of metal content.

The same structural sensitivity is even more pronounced in optical and photothermal systems. The localized surface plasmon response depends directly on particle size, shape, and near-field coupling, while aggregation can shift resonance, broaden spectra, and alter the balance between scattering and absorption. Studies on shape controlled Au nanoparticles, Pt nanoparticles, gold nanorods, and anisotropic plasmonic structures all indicate that optical response is governed by geometry specific resonance and coupling mediated spectral redistribution.^115–122^ This dependence also explains the trade-off between photothermal efficiency and optical transparency. Ordered low coverage networks, shell engineered nanostructures, and confined nanoparticle architectures have shown that these two objectives can be partially reconciled when aggregation is controlled rather than merely suppressed or intensified.^120–124^

Mechanical response follows a related but distinct pathway. Nanoparticles may improve fracture resistance by redistributing local stress and constraining crack propagation, yet architectures that maximize conductivity are not necessarily optimal under repeated deformation. Recent work on hybrid conductive composites and hierarchical stretchable networks shows that mechanically durable systems increasingly rely on local bundling, porous pathways, or other multi-level conductive motifs that preserve transport while reducing strain localization.^125–129^ Morphology therefore controls not only the magnitude of response, but also which functional mode remains dominant under mechanical loading.

### 4.2 Interphase-mediated network formation and percolation-driven transport

In metal nanoparticle–polymer nanocomposites, the interface is more appropriately treated as an interphase with distinct structure, dynamics, and transport consequences. Molecular simulations, interface engineering studies, and surface modification strategies consistently show that polymer chains near nanoparticle surfaces exhibit altered conformation, relaxation behavior, and packing states relative to the bulk matrix.^130–133^ This interphase acts as an effective third phase that modifies local mobility, dielectric polarization, colloidal stability, and interparticle accessibility. As a result, transport depends not only on whether particles are geometrically close, but on whether the interphase permits effective electrical, optical, or chemical coupling.

For this reason, percolation in multifunctional nanocomposites should be interpreted as an interphase mediated network transition rather than as a purely geometric threshold. Experimental and theoretical studies indicate that finite interphase volume, tunneling distance, mesoscale arrangement, and dimensional confinement all shift the onset and stability of transport.^134–136^ In some systems, two stage percolation behavior emerges because electron transport is sustained first by tunneling and later by more continuous conductive pathways. These results confirm that connectivity, accessibility, and barrier state must be considered together.

A related issue concerns the balance between structural stability and controlled reconfigurability. Some interfaces are designed to maximize environmental robustness and suppress drift, whereas others are engineered to enable self-healing or controlled rearrangement under damage.^137,138^ In practice, optimal interphase design depends on service condition, intended failure mode, and whether reversible reconstruction is more valuable than static rigidity.

Recent surface coating strategies have further expanded the design space of interfacial layer-mediated structure–property relationships. In recent studies, the coating layer is no longer regarded merely as a stabilizer for improving dispersibility, but as a tunable functional interfacial layer that regulates the interparticle distance, surface oxidation, matrix compatibility, interfacial adhesion and active surface accessibility of metal nanoparticles. Polymer brushes or grafted shell layers can effectively regulate the miscibility of nanoparticles in polymer matrices and the effective interparticle distance by adjusting the grafting chain length and grafting density, thereby influencing the percolation threshold and mechanical reinforcement behavior. Existing studies have demonstrated that at moderate grafting densities, nanoparticles are more prone to forming a continuous percolation network, which leads to a reduced percolation threshold.^139–141^ Silica shells can stabilize plasmonic nanostructures such as Au and Ag, and inhibit irreversible agglomeration, while the shell thickness will alter their plasmon resonance conditions.^142,143^ Biomimetic coatings such as polydopamine and tannic acid can immobilize metal nanoparticles through strong adhesion and multi-site coordination, regulate Ag^+^ release and achieve long-term stability.^144–146^ Therefore, surface coating should be regarded as a function-oriented interfacial engineering rather than a simple anti-agglomeration treatment.

### 4.3 Coupled multifunctionality and performance trade-offs

A defining characteristic of these nanocomposites is that different responses are structurally coupled rather than independently adjustable. Electrical conductivity, dielectric polarization, photothermal conversion, mechanical deformability, and biological activity often depend on overlapping descriptors, including network continuity, aggregation state, interphase thickness, and accessible surface area. As a result, improving one function frequently perturbs another.

The most established example is the conductivity–deformability trade-off. High conductivity generally requires continuous conductive pathways, whereas stretchable systems must avoid brittle stress concentration and preserve network integrity under strain. Recent strategies based on locally bundled nanowires, mechanically interlocked hydrogel electrodes, liquid metal hybrids, and porous phase separated architectures show that this trade-off can be moderated through hierarchical design rather than high filler loading alone.^128,129,147–149^ A comparable tension exists in optical and photothermal systems, where clustering often enhances heat generation but may reduce transparency, broaden spectra, or accelerate irreversible drift.^123,124,150^ In biomedical systems, the principal trade-off lies between activity and safety. Strong antimicrobial response often requires high surface accessibility or ion release, but those same features may increase cytotoxicity. Cooperative strategies based on polymer regulation, synergistic multi-component systems, and interfacially localized architectures indicate that efficacy is more sustainably improved by structural control than by simple dose intensification.^151–154^ In multifunctional composite materials related to aerospace and defense, metal fillers can enhance mechanical properties and functional strength. However, excessive filling can increase the structural weight of the composite material, thereby undermining the pursuit of ultimate lightweighting in aerospace structures. Moreover, high nanoparticle content often promotes aggregation, resulting in uneven dispersion, which may cause defects or stress concentration points, reduce interface load transfer, and lower mechanical properties.^155–157^ Overall, multifunctionality in these materials is best understood as a constrained multi-objective design problem.

### 4.4 Multiscale characterization and predictive modeling of structure–property relationships

Because the relevant structures span the nanometer, mesoscale, and device scales, structure–property analysis increasingly requires explicitly multiscale methods. Recent reviews and modeling studies show that no single framework can capture local particle arrangement, interphase behavior, mesoscale connectivity, and macroscopic effective properties simultaneously.^158–160^ Microscopy, scattering, and spectroscopy therefore become most useful when interpreted through integrated computational models rather than as isolated descriptors.

Data driven approaches are accelerating this transition from descriptive characterization to predictive design. Machine learning combined with finite element analysis and microstructure informed modeling has already shown that complex structural fields can be linked quantitatively to effective material response.^158–162^ For multifunctional metal nanoparticle–polymer nanocomposites, the main opportunity lies in combining experimentally resolved descriptors, such as spacing, clustering statistics, orientation, and interphase features, with multiscale simulation to construct transferable process–structure–property maps.

Recent mathematical and theoretical studies have greatly enriched our understanding of the development mechanisms of the surface and interface of metal nanoparticle–polymer nanocomposites. Atomic and molecular dynamics simulations have been used to reveal the behavior of polymer chains around bare and core–shell gold nanoparticles,^131^ and a full-atom model of the polymer interface with gold nanoparticles has been constructed,^163^ providing quantitative microscopic mechanism explanations for understanding the properties mediated by the interface. Microscopic mechanical models further link the interface strength and macroscopic mechanical properties, thus expanding the surface/interface analysis from molecular structure to the effective composite material response level.^164^ In addition, theoretical frameworks based on percolation and tunneling effects have clarified how particle spacing, transmission barriers, and connectivity related to the interface control the conductivity of metal nanoparticle–filled polymer systems. Mohammadpour-Haratbar *et al.*^113^ incorporated the key geometric and physical parameters of silver nanowires into the conductivity calculation based on the tunneling percolation theory model, confirming that the volume fraction, aspect ratio, interface thickness, and tunneling distance of the nanowires are the most significant factors affecting the conductivity of the nanocomposite. And by combining numerical methods with Monte Carlo sampling, it can be used to study the electrical conduction mechanism of the gold nanoparticle–polymer system.^135^

### 4.5 Stability, reliability, and environmentally conditioned structure evolution

Long term stability is the decisive test of whether an apparent structure–property relationship is practically meaningful. In multifunctional nanocomposites, performance evolves continuously as interfaces reconstruct, particles migrate, conductive pathways rupture or reform, and plasmonic or catalytic structures coarsen under heat, light, strain, or chemical exposure. Durability should therefore be treated as an extension of structure–property analysis rather than as a separate engineering concern.

Experimental studies support this dynamic view. Mechanical deformation can alter both optical and electrical output by changing interparticle spacing and local coupling, while aging and environmental exposure may progressively rewrite transport pathways through crosslinking, phase separation, oxidation, or interfacial degradation.^147,165–167^ At the same time, dynamic matrix systems, including vitrimer based nanocomposites, suggest that future durable materials may depend not only on passive stability, but also on controlled repairability and structural reconfiguration. High performing metal nanoparticle–polymer nanocomposites are therefore defined less by a single optimal microstructure than by application specific compromises among connectivity, confinement, accessibility, deformability, and resistance to environmental drift.

## 5 Emerging application domains enabled by controllable fabrication and tailored structure–property relationships

**Fig. 6:**
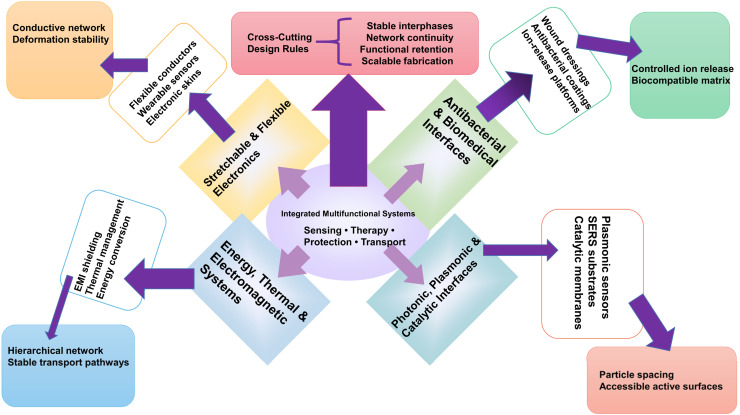
Application specific structural requirements for multifunctional metal nanoparticle–polymer nanocomposites.

The application value of multifunctional metal nanoparticle–polymer nanocomposites depends on whether their internal architectures can be deliberately established and retained under realistic operating conditions. In these materials, performance is governed less by composition alone than by the arrangement of metallic building blocks, the accessibility and stability of the interphase, and the evolution of conductive or reactive networks during service. Recent developments across flexible electronics, sensing, biomedical interfaces, and energy related systems therefore show that controllable fabrication is no longer simply a route to better laboratory structures, but a prerequisite for device oriented materials design (Fig. 6).

### 5.1 Stretchable conductive networks for flexible electronics

Flexible and stretchable electronics require more than high conductivity. The essential requirement is the preservation of stable conductive pathways under repeated bending, stretching, and cyclic deformation. This has shifted materials design away from maximizing filler loading and toward engineering conductive architectures capable of redistributing strain or reconstructing transport pathways during deformation.^168–171^ Recent studies on low loading conductive nanocomposites, printable viscoelastic conductors, honeycomb networks, and anisotropic freeze dried structures show that electrical continuity and mechanical compliance can be better balanced through topology control than through dense metallic filling.^170–174^ In this application domain, the decisive descriptor is therefore conductivity retention under deformation rather than conductivity alone.

### 5.2 Wearable sensing platforms and human–machine interfaces

Wearable sensing imposes a different requirement. Here, the goal is not to minimize resistance variation, but to convert small structural perturbations into stable and interpretable electrical signals. Metal nanoparticle–polymer nanocomposites are particularly well suited to this task because their transport behavior is highly sensitive to interparticle spacing, contact resistance, and the reversible disruption of local conductive junctions.^175,176^ Sponge like conductive frameworks, elastomer based piezoresistive systems, fibrous hybrid networks, coated textiles, and three dimensional core–shell architectures all show that high sensitivity typically arises from deliberately engineered structural heterogeneity rather than ideal uniformity.^177–182^ From a structure–property standpoint, the central design principle is controlled near criticality: the network must be sufficiently responsive to mechanical input, yet sufficiently stabilized to avoid catastrophic drift.

### 5.3 Antibacterial and biomedical interfaces

In antibacterial and biomedical systems, functional effectiveness must be balanced against biocompatibility, release control, long term stability, and regulatory reproducibility. Metal nanoparticles offer strong antimicrobial and therapeutic potential, but the same features that enhance activity may also increase cytotoxicity or uncontrolled exposure. The central problem is therefore not simply whether metallic particles are incorporated, but how their distribution, interfacial accessibility, and release behavior are regulated within the polymer matrix.^183–185^ Recent work on antimicrobial nanocomposites, therapeutic metallic systems, silver containing scaffolds, pH responsive nanoparticle hybrids, and bimetallic antibacterial systems indicates that the most promising route is not unrestricted nanoparticle exposure, but structurally localized, compositionally tuned, and release regulated interfaces.^186–188^ In biomedical applications, the matrix and interphase are therefore inseparable from the metal phase in determining performance.

### 5.4 Energy conversion, thermal management, electromagnetic shielding, and catalysis

In energy and electromagnetic applications, the advantage of metal nanoparticle–polymer nanocomposites lies in the combination of low density and processability with conductive, thermal, or catalytic functionality. Relative to many polymer nanocomposites based on insulating ceramic, oxide, or polymeric fillers, metallic nanocomposites offer a more direct route to coupled charge transport, heat dissipation, and interfacial activity. Percolated Ag networks in epoxy convert an insulating matrix into a conductive composite,^189,190^ while Cu nanoparticles or Cu nanowires in PMMA enhance thermal transport by forming high-conductivity heat-transfer pathways.^191,192^ When polymer–metal interactions are strong, metallic nanoparticles can also reinforce the matrix, as exemplified by Ag/chitosan hybrid fibers in which chemical interactions between Ag nanoparticles and nitrogen-containing groups and glycosidic oxygen atoms in chitosan enhance the mechanical properties of the fibers.^193^ These coupled mechanical, thermal, and electrical advantages are particularly valuable in lightweight and processable materials for energy conversion, thermal management, electromagnetic shielding, and catalysis.

These performance gains also carry practical implications for cost and energy efficiency. Metal-containing polymer shields can replace heavier bulk-metal enclosures, reducing weight and metal consumption without sacrificing shielding effectiveness.^194^. In thermal-management components, enhanced heat spreading suppresses local hot spots and can reduce cooling requirements.^195^ In catalytic systems, polymer-supported metal nanoparticles improve dispersion, stabilize accessible active sites, and facilitate recovery and reuse, thereby limiting noble-metal loss and lowering total cost.^196^

Yet high metallic content alone does not guarantee useful performance. What matters instead is whether conductive pathways, thermally active domains, or catalytic interfaces are organized into accessible and stable mesoscale architectures.^173,197–200^ Dual-network foams, stretchable shielding textiles, nanofibrous electrochemical supports, and polymer–metal catalytic interfaces all demonstrate that device-level response emerges only when transport efficiency, network stability, and interfacial accessibility are co-designed.^197–200^ Thus, in this application domain, performance is interface-dominated rather than composition-dominated.

### 5.5 Toward integrated and application specific multifunctional systems

A clear trend across recent work is the convergence of multiple functions within a single structurally engineered platform. Flexible electronics increasingly overlap with sensing and thermal management, wearable systems require conductivity together with softness and durability, and biomedical materials are expected to combine bioactivity with structural support and safety.^168,175,182,183,200^ Under these conditions, fabrication route, network topology, and operating environment must be co-optimized from the outset. Printing, freeze drying, *in situ* assembly, coating, and interphase modification are therefore not interchangeable processing routes, but distinct ways of distributing conductive, responsive, or bioactive elements in space. The key challenge is no longer whether individual functions can be achieved, but whether they can be spatially coordinated and retained within a single device relevant architecture.

### 5.6 Representative case studies linking fabrication to device level performance

Across current application domains, representative studies support the same process–structure–property–application logic. In stretchable conductors, printable viscoelastic systems and anisotropically organized nanocomposites show that controlled network rearrangement and topology design are more important than rigid conductive density for maintaining electrical performance under strain 171,174. In wearable sensing, porous sponges, fibrous hybrid networks, textile based coatings, and three dimensional sensor architectures confirm that signal amplification depends on structurally sensitive yet mechanically sustainable conductive regimes.^177,179–182^ In energy and protective materials, dual network foams and multifunctional shielding fabrics show that device level performance emerges from the coordinated design of conductive pathways, interfacial chemistry, and mesoscale geometry.^197,200^ In antibacterial systems, polymer regulated silver scaffolds and Ag–Cu cooperative systems further demonstrate that fabrication controlled composition, distribution, and release can convert nanoscale synergy into biologically meaningful function while limiting uncontrolled exposure.^186,188^ Taken together, these case studies indicate that high performance application systems are not defined by any single optimal parameter, but by the successful alignment of fabrication route with the structural features that govern function under real service conditions.

## 6 Conclusions, current limitations, and future perspectives

Metal nanoparticle–polymer nanocomposites have evolved from simple filler-containing blends into structurally programmable hybrid material systems capable of integrating electrical, optical, thermal, catalytic, antimicrobial, magnetic, and electromagnetic functions within lightweight and processable polymer matrices. A central conclusion of this review is that their macroscopic performance is not determined solely by metal type or filler loading. Rather, it is governed by a set of coupled structural descriptors, including nanoparticle morphology, dispersion state, interparticle spacing, interphase chemistry, network topology, and structural evolution under service conditions. A descriptor-based perspective is therefore essential for transforming empirical formulations into rational materials design.

Despite substantial progress, several limitations still hinder practical translation. Quantitative structure–property relationships remain insufficiently established, partly because key descriptors are not reported in a consistent and standardized manner across different studies. Long-term reliability is another major challenge. Mechanical deformation, thermal cycling, humidity, irradiation, oxidation, swelling, and exposure to chemical media may induce nanoparticle migration, coarsening, detachment, or network reorganization, leading to functional drift. In addition, scalable manufacturing often introduces structural heterogeneity that is not evident in small laboratory specimens, thereby reducing reproducibility and complicating process control. For applications involving direct biological or environmental exposure, safety and sustainability issues also remain incompletely resolved.

Looking ahead, further progress will increasingly depend on function-oriented interphase engineering rather than generalized particle-stabilization strategies. Surface coatings, grafted layers, and hybrid interphases should be designed according to the dominant transport or reaction mechanism of the target application, so that stability, accessibility, and performance can be optimized in a coordinated manner. At the same time, theoretical and data-driven approaches are expected to play a more important role in guiding surface and structural design, particularly when precise multiscale control over interparticle spacing, barrier characteristics, local dielectric response, and network connectivity is required.

Several directions are especially important for the next stage of development. First, descriptor-based reporting should be further standardized to improve comparability across material systems and processing routes. Second, scalable fabrication should be coupled with quality-control strategies capable of preserving nanoscale dispersion and mesoscale transport-pathway continuity during manufacturing. Third, multifunctional performance should be evaluated using application-oriented metrics rather than isolated peak values. Finally, safety-by-design and sustainability considerations should be incorporated from the early stages of materials development, especially for biomedical, wearable, packaging, and environmental applications.

Overall, the most promising metal nanoparticle–polymer nanocomposites will not be those with the highest metal content, but those that use a minimal metallic phase to construct stable, accessible, and hierarchically connected functional pathways. Achieving this goal will require the integration of interphase engineering, descriptor-based characterization, theory-guided design, scalable processing, and standardized evaluation.

## Conflicts of interest

There is no conflict of interest.

## Data Availability

No primary research results, software or code have been included and no new data were generated or analyzed as part of this review.
